# Facial cues to age perception using three-dimensional analysis

**DOI:** 10.1371/journal.pone.0209639

**Published:** 2019-02-13

**Authors:** Takeo Imai, Kyoko Okami

**Affiliations:** Skin Care Laboratory, Kao Corporation, Odawara, Kanagawa, Japan; UNITED KINGDOM

## Abstract

To clarify cues for age perception, the three-dimensional head and face forms of Japanese women were analyzed. It is known that age-related transformations are mainly caused by changes in soft tissue during adulthood. A homologous polygon model was created by fitting template meshes to each study participant to obtain three-dimensional data for analyzing whole head and face forms. Using principal component analysis of the vertices coordinates of these models, 26 principal components were extracted (contribution ratios >0.5%), which accounted for more than 90% of the total variance. Among the principal components, five had a significant correlation with the perceived ages of the participants (*p* < 0.05). Transformations with these principal components in the age-related direction produced aged faces. Moreover, the older the perceived age, the larger the ratio of age-manifesting participants, namely participants who had one or more age-related principal component score greater than +1.0 σ in the age-related direction. Therefore, these five principal components were regarded as aging factors. A cluster analysis of the five aging factors revealed that all of the participants fell into one of four groups, meaning that specific combinations of factors could be used as cues for age perception in each group. These results suggest that Japanese women can be classified into four groups according to age-related transformations of soft tissue in the face.

## Introduction

All humans are destined to grow old, but a youthful and beautiful figure is something that everyone longs for. A considerable number of approaches involving non-operative procedures in the fields of beauty and aesthetics have been taken to identify keys for maintaining a youthful appearance [1, 2]. However, none of these approaches have been shown to play a decisive role in facial antiaging. Although facial appearance varies widely among individuals, some common keys to age estimation are likely embedded in facial features. In order to develop symptomatic and/or preventive solutions in the beauty and aesthetic area, it is therefore essential to clarify changes in individual aging paths.

The judgment of another person’s age by appearance, namely age perception, is important for human beings to achieve good communication and build smooth personal relationships. Faces have a critically important function as a source of biological and social information [3]. Of the numerous aspects of human appearance, facial appearance provides the most essential information about age [4]. “Cardioidal strain,” which describes the age-related craniofacial transformation of the head and face, has been reported as a cue to age perception during the growth period [5]. Since the head and face transform remarkably in that an increase in the size of the face relative to the head takes place during this period, we can recognize differences in age from the outline of an individual’s profile alone [4]. In contrast to the first 20 years of life, the transformations of the cranium during adulthood are few compared with those of the soft tissue [6]. Therefore, the appearance of facial aging is mainly caused by attrition and the deterioration of soft tissue [7, 8]. In the last 40 years, a considerable number of studies have been conducted on the age perception of faces [9]. There are two cues to age perception in adulthood: the first is shape cues (configuration of spatial features), and the other is surface cues (skin texture). Skin texture includes not only facial wrinkling [10], but also skin color distribution [11] and facial contrast [12, 13], all of which affect age perception. It is noteworthy that age perception can be quite robust with respect to shape cues. Experimental manipulations that severely impair texture information, such as negation and blurring of facial images, have relatively little effect on age perception; in other words, age perception is primarily based on shape cues [14, 15].

In the last 20 years, numerous studies have been conducted on methods to age facial images digitally [16, 17]. In the late 1990s, several studies attempted to extract age-related features using statistical methods such as two-dimensional (2D) averaged faces in two age categories (25–29 years and 50–54 years) [18] and 2D facial images of a broad age range (10–70 years), which were analyzed by applying a three-dimensional (3D) facial-shape polygon model based on principal component analysis (PCA) [19]. These attempts were followed by the application of statistical methods using linear and quadratic modeling in the PCA space [20, 21]. Since these works were based not only on inter-individual data, but also on intra-individual age-progressive data, they discussed person-specific aging paths. However, the databases used in these studies were built with relatively young facial images (5–30 years [20] and childhood to youth [21]); therefore, most of the transformations included in these databases corresponded to the participants’ growth periods.

These previous studies used 2D facial images; however, approaches currently exist for synthesizing the effects of aging on facial images using 3D polygon modeling. A high-resolution morphable polygon model for synthesizing 3D faces has been proposed for facial manipulation [22]. This polygon model was applied to age the face models using partial least squares regression [23]. A statistical analysis of 3D forms measured using the polygon model to simulate rejuvenated and aged faces has also been reported [24]. Although the above two studies are notable for their discussion of aging paths specific to individuals, they were based on narrow age ranges: from preschoolers to students (5–23 years) [23], children to teenagers, and young adults [24]. These two studies were appropriate for simulating facial images during the growth period, in which the major part of transformations derives mainly from the transformation of the cranium.

The studies that used 2D facial images to create 3D models based on a broad age range (from 10–70 years [19] and from 0 (less than 12 months) to 69 years [25]) created aged facial images, but the depth direction (anteroposterior) component had to be approximated for the fitment to the facial form. Although these methods were appropriate for reproducing the contour, size, and configuration of facial parts, they were not ideal for reproducing the prominences and/or hollows around the grooves and bags in the cheek and lower jaw areas. Although the above studies analyzed 3D forms and textures, little attention has been given to statistical extraction of age-related facial transformations in soft tissue using a 3D facial form database with a broad age range.

Previously, 3D form measurements were used to evaluate partial transformations of the face area [26–28]. Although some studies have statistically analyzed 3D facial forms, these were conducted for anthropometric analysis [29] and to examine the link between perceived and measured face shapes [30]. To our knowledge, no studies have attempted to analyze age perception from the viewpoint of the whole 3D craniofacial form. Statistical analyses and simulation methods have been proposed for variations in the human body form using homologous models [31], and this model has been applied to predict up to 30 years of facial aging in an average Japanese male [32].

In the present study, our intention was to build a whole head and face database with a broad age range to clarify aging transformations specific to facial appearance, such as grooves and bags from and after middle age. Since human facial features vary from person to person, landmarking has been accepted as a general method for data representation [33, 34]. To detect delicate transformations in soft tissue, each 3D polygon model was fitted directly to 3D measured data using landmarks. Compared with other computer models for the human head, the homologous polygon model using landmarks provided meaningful statistical results by guaranteeing that head and face forms were the same for all participants. An additional benefit was the preservation of more landmark information, which was useful for further analysis of form variations in the areas around the landmarks [35].

Differences between the sexes in regard to age-related transformation in the head and face area have been reported [36]. To exclude the difference between the sexes, we first established a database of Japanese women, because the aim of the present study was to clarify the cues leading to age perception through the creation of a database comprising whole 3D head and face forms of real Japanese women of various ages. Next, to clarify the aging patterns, we classified the participants according to aging changes. The database was then statistically analyzed using homologous polygon models to explore actual whole facial aging paths and classification by specific age-related transformations.

## Materials and methods

### 1. Ethics statement

This study followed ethical protocols in accordance with the principles expressed in the Declaration of Helsinki and the guidelines of the Ethical Committee for Human Research of Kao Corporation (Tokyo, Japan). The rules of this committee are reported to the Japan Agency for Medical Research and Development’s Research Ethics Review Committee Report system (IRB No. 17000058). These guidelines include the signing of informed consent forms by the study participants for participation in the study, for being photographed, and for somatometric measurements of their head and face area. The voluntary nature of participation in the study was explained to all of the participants, including an explanation that they would not be compelled to complete the examination if they refused or interrupted it at any time, and that there would not be any retaliation or sanctions against them. The participants were paid a fixed amount for their participation in this study. Personally identifiable data, such as addresses and names, were not collected in the interest of protecting privacy. Furthermore, photographs and 3D measurement data of the head and face area, by which participants could possibly be identified by others, were registered and placed under control according to the Personal Information Guideline of Kao Corporation (registration No. 1200166).

### 2. Participants

The study participants were 148 adult Asian females of Japanese nationality aged 20–69 years at the start of the study (mean biological age = 44.3 years). In the present study, we use the term “Japanese women” to refer to Asian females of Japanese nationality. The number of participants by age group was as follows: 29 (20s), 29 (30s), 30 (40s), 30 (50s), and 30 (60s).

The inclusion criteria were being a healthy Japanese women and providing written, informed consent. Those with a cutaneous pathology in the head and face area, such as eczema, were excluded.

### 3. Age perception

The ages of the participants were estimated from facial images presented on a 518.4 × 324.0 mm color-calibrated LCD monitor (ColorEdge CG242W; EIZO Corporation, Hakusan, Japan) that was kept at a distance of about 70 cm from the raters. The stimuli were two facial images of each participant photographed under the same lighting conditions, with identical frontal and rotated [30°, left side] views, with natural facial expressions and no makeup, and with their clothes digitally blocked over in gray using general purpose software. The two facial images from each participant, each about 15 cm in size and separated by 5 cm, were presented simultaneously. To counteract the effect of skin color distribution on age estimation, the stimuli were shown in 256-level gray scale color. In addition, the stimuli were shown in predetermined random sequences to help prevent any bias in age estimation caused by viewing the previous images. Each rater was requested to estimate the age of the faces using a single-year step scale range. The exposure time of stimuli was not fixed, and the mean exposure time of all raters was 15.1 s per participant (standard deviation [SD] = 3.0 s).

A previous study found that the accuracy of the raters’ age estimations decreased when the raters performed age estimation of unfamiliar generations [37]. We therefore utilized professional female hair stylists and makeup artists who often met with women of various ages. Ten professional female hair and makeup stylists in their 30s to early 50s (mean biological age = 39.9, SD = 6.8) served as raters for the age estimation test. Each rater signed a confidentiality agreement concerning privacy protection of the participants. The raters judged the ages of the participants from photographs, and their perceived ages were derived by calculating the average of the age estimates.

Next, we tested the internal consistency of the age estimates and the influence of the generation of the participants on age estimation. The internal consistency regarding the estimated ages was calculated using Cronbach’s alpha. The coefficient was sufficiently high (α = 0.94), and the (α) coefficients were 0.79, 0.85, 0.82, 0.90 and 0.90 for the 20s, 30s, 40s, 50s, and 60s age groups, respectively. In addition, the average accuracy in estimated ages was calculated using Pearson product-moment correlation coefficients (Pearson’s *r*) between the estimated and actual ages [38]. The relationship between these ages was sufficiently high (*r* = 0.95). Therefore, the reliability of the age estimation was considered suitable for the purposes of the present study. Although only a small number of raters estimated the age of the faces, their ratings were consistent. To check the influence of the participants’ generations, we calculated the accuracy of the perceived ages for different participant age groups. A one-way analysis of variance (ANOVA) was used to compare the differences between the biological and perceived ages of the participants between biological age groups (five categories from the 20s through the 60s). No significant differences in age estimation accuracy between the biological age groups was observed (*F*(4, 143) = 0.63, *p* = 0.64 [*n*.*s*.]). In spite of the relatively narrow age range of the raters, the age estimations for each age group were accurate, indicating that the test results support the use of perceived ages in the present study.

### 4. Analysis of head and face form

Head and face forms were analyzed according to the following procedure: (i) measure the head and face form of each participant using a 3D digitizer; (ii) obtain homologous polygon models by fitting template meshes; and (iii) analyze the homologous models using PCA and extract age-related principal components (PCs).

#### 4–1 Three-dimensional (3D) measurement of head and face area

**Fig 1:**
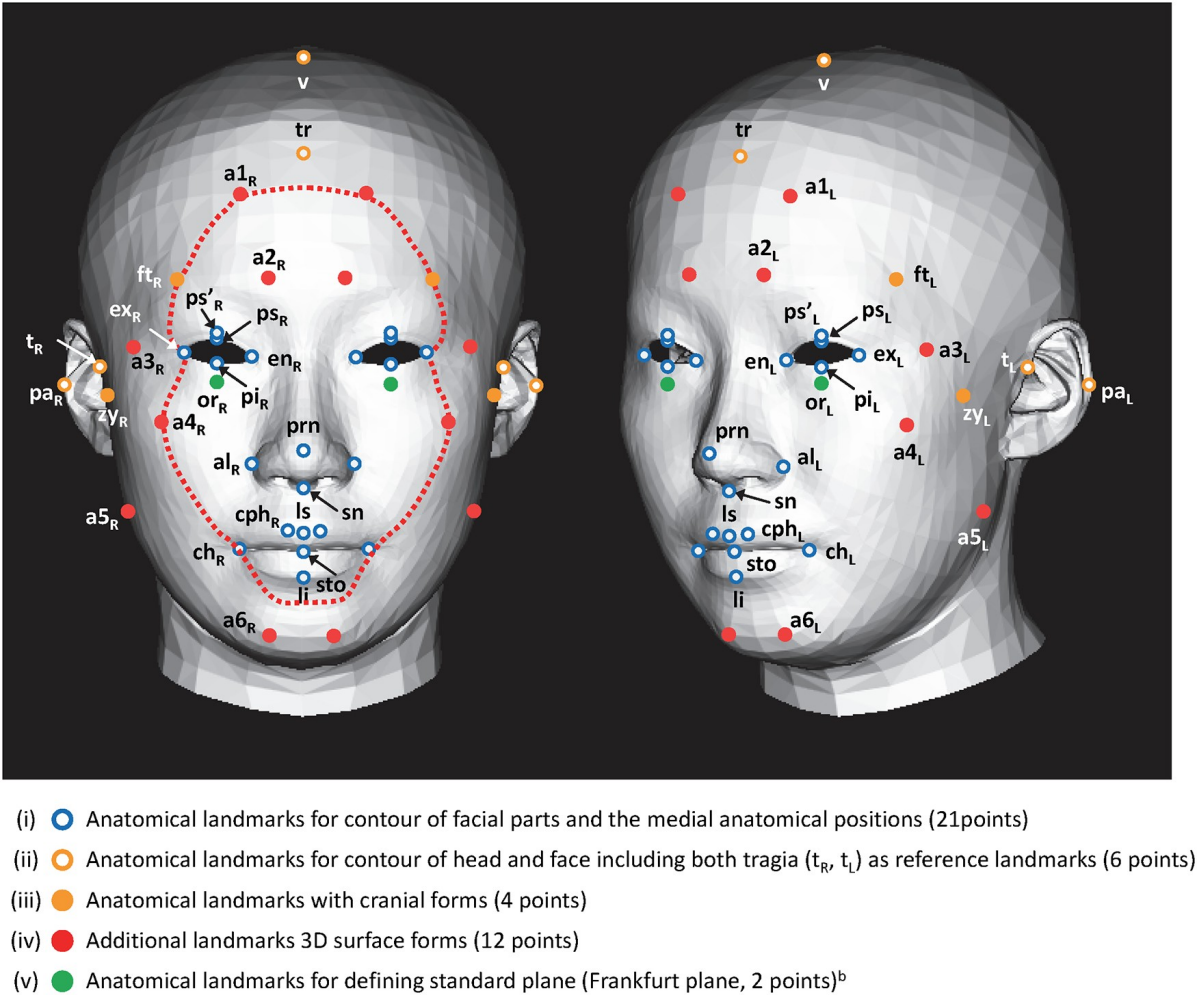
Landmarks for polygon modeling^a^. ^a^ Identical frontal and rotated [30°, left side] views of polygon models (averaged form) used in the present study. The abbreviations for the right and medial landmarks were indicated in the frontal view, and those for the left and the medial landmarks were indicated in the rotated view of the polygon models. The red dotted line represents a contour line in the *z*-axis that passes along both ectocanthions (lateral corners of the eyes, see Supporting Information 1–2) to describe the locations of the landmarks a4_R_ and a4_L_. This line was not used in the analysis. ^b^ These landmarks were not used to create the homologous polygon models.

After marking several landmark locations with stickers, each participant donned a net cap for measurement using a VIVID 910 noncontact-type digitizer (Konica Minolta Sensing, Inc., Osaka, Japan). A Micro Scribe-G2 contact-type digitizer (Revware, Inc., Raleigh, NC, USA) was used to measure the head area to avoid any influence from hair volume. We measured 16 to 24 coordinates, including the following landmark locations: vertex (v), euryons (right and left sides [R/L]), and opisthocranion (see S1 Table). The two data measurements were then integrated using 3D-Rugle software (Medic Engineering, Inc., Kyoto, Japan). The Frankfurt plane [39], which is defined as the plane that passes through two tragia (t) and one orbitale (or), was used as the anatomical reference position of the head and face area, and the origin of the coordinates was located at the midpoint of the line joining both tragia (Fig 1). The *x*-axis is the lateral axis of the frontal face, the *y*-axis is the vertical axis (craniocaudal axis), and the *z*-axis is the depth axis (anteroposterior axis).

#### 4–2 Landmarks of head and face area

Landmarks for the contour of the face and facial parts have been applied to evaluate the relationship between facial features and impressions using 2D facial images [40–43]. On the other hand, landmarks for the contour of facial parts and medial anatomical positions have been used to study 3D facial morphology focusing on soft tissue [44, 45]. These anatomical landmarks are useful for analyzing the contour, as well as the configuration and size of the face and facial parts. In addition to these landmarks, we used several anatomical cranial form landmarks [46] to analyze whole head and face forms.

Homologous polygon models of the head and face area were created by fitting template meshes onto the integrated individual data of all participants using the set of landmarks. The vertices of the polygon meshes were fitted homogeneously, except for the vertices that corresponded to landmarks; these were selectively fitted to the corresponding landmarks. To extract the factors that dictate the impression of aging, the landmarks were used to create homologous polygon models. To analyze the 3D surface forms, including soft tissue, which is closely related to how impressions of aging are formed, we introduced several additional landmarks apart from the general anatomical landmarks. The inflection points on facial surfaces, such as the most prominent point in each area and the saddle points, were also applied. By contrast, we removed several medial anatomical landmarks that were too close to the additional ones to avoid interactions between adjacent landmarks. Finally, as shown in Fig 1, the following 45 landmarks were used: (i) the anatomical landmarks that represent the outline of facial parts and the medial anatomical positions (21 points, open blue circles); (ii) the landmarks that represent the contour of head and face, including both tragia (t_R_, t_L_) as reference landmarks (6 points, open orange circles); (iii) the anatomical landmarks with cranial forms (4 points, closed green circles); (iv) the additional landmarks for 3D surface forms, which are closely related to soft tissue transformations that occur in adulthood (12 points, closed red circles); and (v) the anatomical landmarks for defining the standard plane, which were not used to create homologous polygon models (2 points, closed blue circles). The landmarks used to create the homologous polygon models are described in S2 Table.

Although numerous studies have been conducted to locate 3D features automatically, the accuracy and reliability of those methods have not been confirmed [47, 48]. Landmarks (iii) and (v) were located by palpation and manually marked by stickers before the 3D measurements. The coordinates of all landmarks were manually positioned using 3D-Rugle software (Medic Engineering, Inc.).

#### 4–3 Polygon modeling and statistical analysis of head and face forms

**Fig 2:**
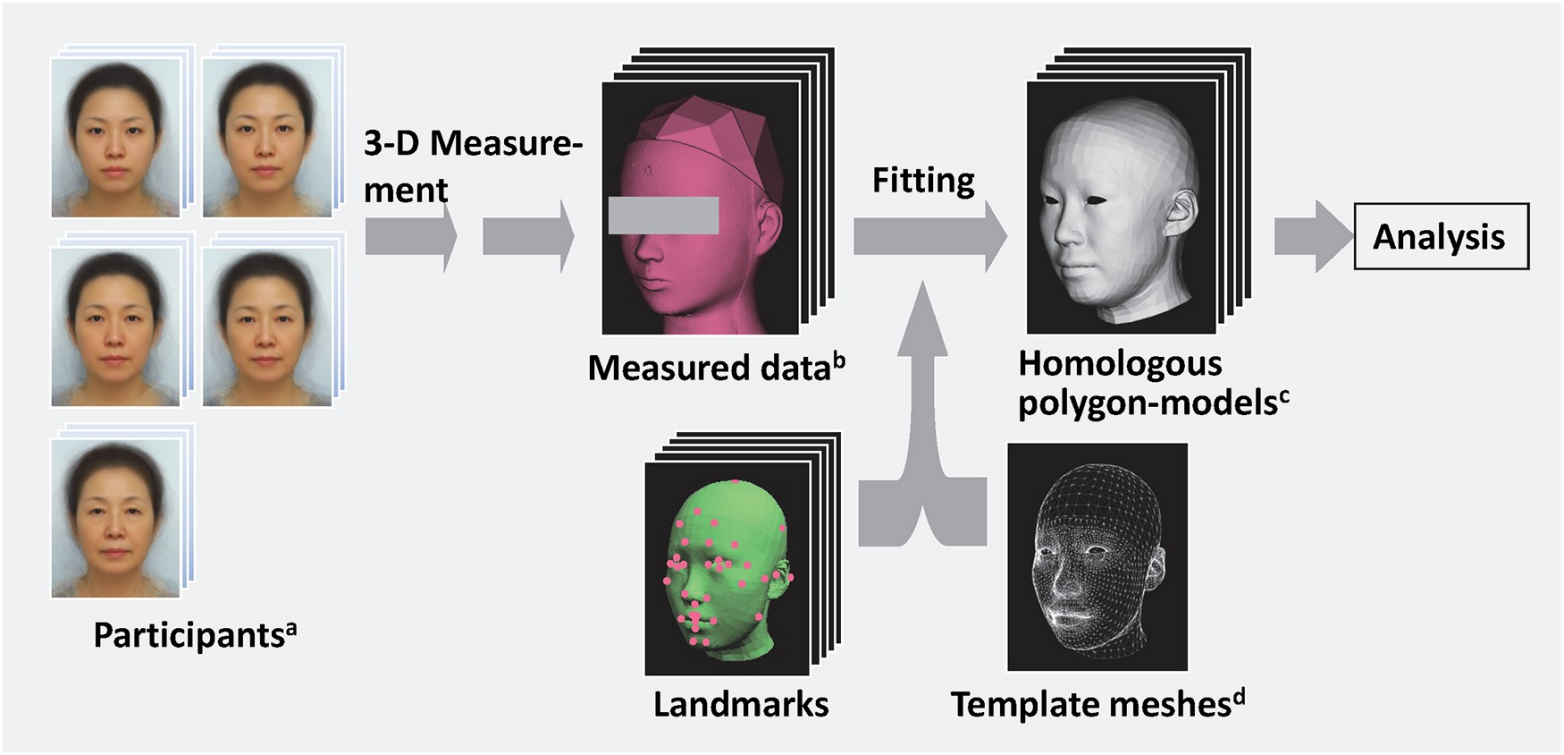
Schematic illustration of the three-dimensional (3D) measurements and homologous polygon modeling. ^a^ Images of averaged faces were used instead of actual participant images in this schematic illustration. ^b^ Measured data were obtained from a mannequin head instead of actual participants. ^c^ The average form of all participants was used instead of homologous polygon models of actual participants. ^d^ The Dhaiba Model was used for the template meshes.

A homologous polygon model was created for each participant by fitting the template meshes to the integrated individual data using the coordinates of the 43 landmarks (Fig 2). The Dhaiba Model (Digital Human Technology Inc., Yokohama, Japan) was used for the template meshes [49], and Homologous Body Modeling software (Digital Human Technology Inc.) was used to create the homologous polygon models. There were 4,703 vertices in the template meshes. The landmarks were then used to fit the template meshes on the 3D form of each participant. To create the homologous polygon models with the same topology, each landmark was aligned on the same vertex of the template meshes. The vertices coordinates of the polygon models were analyzed using PCA [50]. Homologous Body Statistica software (Digital Human Technology Inc.) was used for the PCA. Simulated polygon models of the head and face form *x* were obtained using the following Eq (1):

$$x=\sum_{j=1}^{n}a_{j}v_{j}+m$$ (1)

where ***a***_***j***_ is the ***j*** th principal component score, ***v***_***j***_ is the ***j*** th eigenvector, ***n*** is the number of principal components used, and ***m*** is the average form.

#### 4–4 Relationship between perceived age and PCs of the polygon models

To extract variations in age-related forms, we calculated Pearson’s *r*s and the *p*-values between the perceived ages of the participants and the scores of the 26 PCs. The *p*-values were calculated using a two-tailed significance test, followed by the Benjamini–Hochberg (BH) procedure [51] for multiple testing corrections. The number of tests (*n*) was set to the number of PCs examined, and the false discovery rate (*q*) was set to 0.05 unless stated otherwise.

#### 4–5 Perceived age of simulated polygon models

The previous section describes the method used to estimate ages. Five of the 10 raters estimated the perceived age of the simulated polygon models for each of the participants. The stimuli were images of simulated polygon models (rotated 30°, left side) of the seven obtained polygon models (–3.0 σ, –2.0 σ, –1.0 σ, ±0.0 σ, +1.0 σ, +2.0 σ and +3.0 σ) for each PC. These images were presented simultaneously 5 cm apart in a double line, and the size of the polygon images was 10 cm. The stimuli were shown in a predetermined random arrangement to avoid influencing age estimations based on positional relationships.

#### 4–6 Classification of participants by age-related PCs

The participants were classified according to age-related PCs using cluster analysis (Ward’s method) to gain a better understanding of the differences in aging transformations.

Multiple regression analysis, with perceived age as the dependent variable and the PC scores of the age-related PCs as the independent variables, was conducted to identify the age-related PCs that play an important role in age perception for each group. The PCs are orthogonal because they are the eigenvectors of the covariance matrix, indicating no multicollinearity problems in these cases.

#### 4–7 Measurement and visualization of the differences between two simulated polygon models

The distances between two simulated polygon models were represented by the distance between corresponding areas of two polygons and measured and visualized by 3D-Rugle software (Medic Engineering, Inc.). The distance between two polygon models was indicated as follows: (1) the reddish area: the distance from the first to the second polygon model in the same *x*–*y* plane coordinates was larger than 1.0 mm on the *z*-axis, namely, the second polygon was interiorly located in this area; (2) the white area: the distance was between 1.0 mm and 0.0 mm; and (3) the bluish area: the distance was less than 0.0 mm, namely, the second polygon was exteriorly located in this area. In addition, the colors became denser with increasing distance.

## Results and discussion

### 1. Analysis of 3D head and face forms with homologous polygon models

Although the cranial form has a fundamental influence on facial features, the principal transformations in adulthood are caused by changes in soft tissue, which appear to be strongly related to how impressions of aging are formed [6–8]. In this study, we focused on the 3D forms of soft tissue to extract the factors that influence the impression of aging.

We used PCs to represent variations in head and face forms [33]. The homologous polygon models of participants (with the landmark set applied) were analyzed using PCA, which resulted in the extraction of 26 PCs (contribution ratios > 0.5%) and accounted for more than 90% of the total variance (see Table 1). The PCs were assigned according to variations in the head and face forms. The first few PCs with larger contribution ratios corresponded to relatively large transformations in the head and face area. These PCs could be assigned by applying somatometric measurements as follows: variations in face size (1st PC), face height (2nd PC), facial angle (3rd PC), head and face breadth (4th PC), head size (5th PC), and head breadth (6th PC). By contrast, the subsequent PCs corresponded to variations in the soft tissue of specific areas, partial transformations, and asymmetrical properties in the head and face area, including variations in the breadth of the mouth (12th PC), the height of the lower face (16th PC), or the head area (11th PC).

**Table 1:** Principal component analysis (PCA) of the homologous polygon models, Pearson’s *r*s between perceived ages, and principal component (PC) scores.

Principal component	Contribution ratio (%)	Cumulative contribution ratio (%)	Pearson’s *r*	*p*-value^a^	Significance^b^
1st	19.70	19.70	0.25	0.004	*p* < 0.05
2nd	12.89	32.59	0.01	0.910	*n*.*s*.
3rd	8.06	40.65	0.07	0.363	*n*.*s*.
4th	7.05	47.70	–0.08	0.308	*n*.*s*.
5th	6.66	54.37	0.16	0.041	*n*.*s*.
6th	5.54	59.91	–0.06	0.375	*n*.*s*.
7th	3.85	63.76	–0.20	0.016	*n*.*s*.
8th	3.58	67.34	0.08	0.368	*n*.*s*.
9th	2.99	70.33	–0.36	< 0.001	*p* < 0.05
10th	2.78	73.11	–0.38	< 0.001	*p* < 0.05
11th	2.34	75.44	0.12	0.207	*n*.*s*.
12th	1.96	77.40	0.31	< 0.001	*p* < 0.05
13th	1.81	79.21	–0.05	0.525	*n*.*s*.
14th	1.69	80.89	–0.03	0.638	*n*.*s*.
15th	1.50	82.39	0.02	0.931	*n*.*s*.
16th	1.22	83.61	0.01	0.908	*n*.*s*.
17th	1.07	84.69	–0.03	0.690	*n*.*s*.
18th	0.94	85.63	0.11	0.192	*n*.*s*.
19th	0.85	86.48	–0.11	0.201	*n*.*s*.
20th	0.84	87.32	0.27	0.001	*p* < 0.05
21st	0.75	88.06	0.11	0.196	*n*.*s*.
22nd	0.72	88.78	–0.16	0.063	*n*.*s*.
23rd	0.67	89.45	0.14	0.139	*n*.*s*.
24th	0.62	90.07	–0.10	0.233	*n*.*s*.
25th	0.55	90.62	0.10	0.212	*n*.*s*.
26th	0.50	91.12	–0.12	0.120	*n*.*s*.

^a^ Actual *p*-values before multiple testing corrections.

^b^ Benjamini-Hochberg procedure (BH procedure) was performed for multiple testing corrections (*q* = 0.05, *n* = 26).

To extract age-related variations in form, the correlation between perceived ages and the scores for each PC were calculated (Pearson’s *r*, multiple testing corrections: BH procedure). The results showed that the following five PCs had significant correlations with perceived age (*p* < 0.05): the 1st (Pearson’s *r* = 0.24), 9th (*r* = –0.36), 10th (*r* = –0.39), 12th (*r* = 0.31) and 20th (*r* = 0.26). The contribution ratio of each PC was not very large (less than 3.0%), except for that of the 1st PC (19.7%).

### 2. Key morphologic factors for age perception

**Fig 3:**
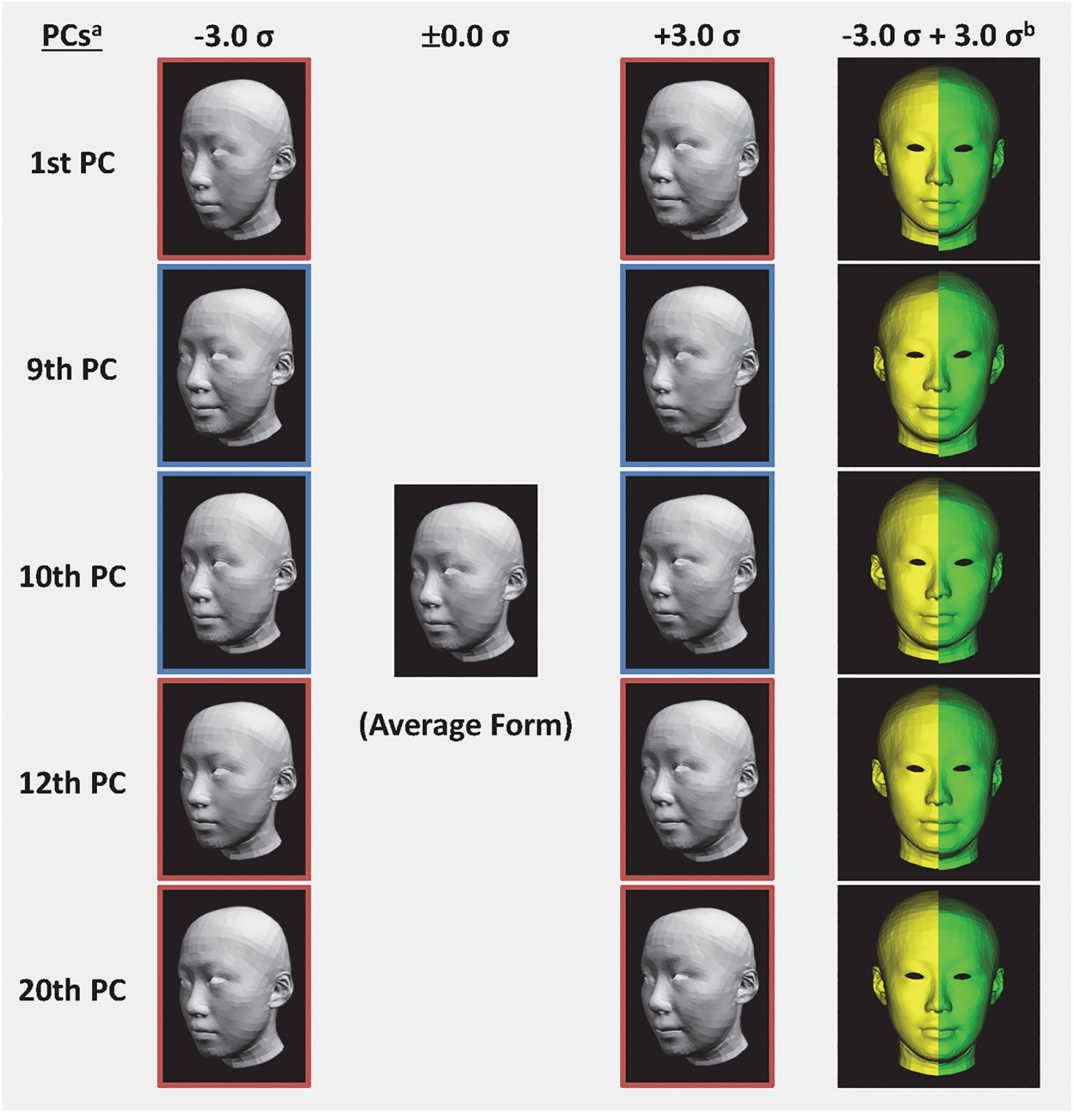
Simulated forms that reflected age-related principal components (PCs). a Each normalized PC score varied from −3.0 σ to +3.0 σ. The 1st, 12th, and 20th PCs were in direct proportion to perceived age (red borders), and the 9th and 10th PCs were in inverse proportion to perceived age (blue borders). b Frontal view image of simulated form (−1.0 σ and +1.0 σ for the 1st PC, −3.0 σ and +3.0 σ for the 9th, 10th, and 12th PCs).

Simulation models were created to visualize transformations owing to age-related PCs. Each normalized PC score varied from –3.0 σ to +3.0 σ. A simulated model with ±0.0 σ corresponds to the average form of the participants (Fig 3).

The 1st PC, which had the largest contribution ratio, corresponded to variations in face size. By contrast, the other PCs, which had small contribution ratios, were classified as being associated with relatively partial transformations of soft tissue, such as variations in the size and/or form of parts of the face. The transformations of these five PCs corresponded mainly to the following form variations: mid and lower face size (1st PC), eye angle and mouth breadth (9th PC), biectocanthion breadth (the distance between both lateral corners of the eyes), eye length and cheek breadth (10th PC), eye angle and mouth breadth (12th PC), and height between the nose and mouth and size of the eyes (20th PC).

**Fig 4:**
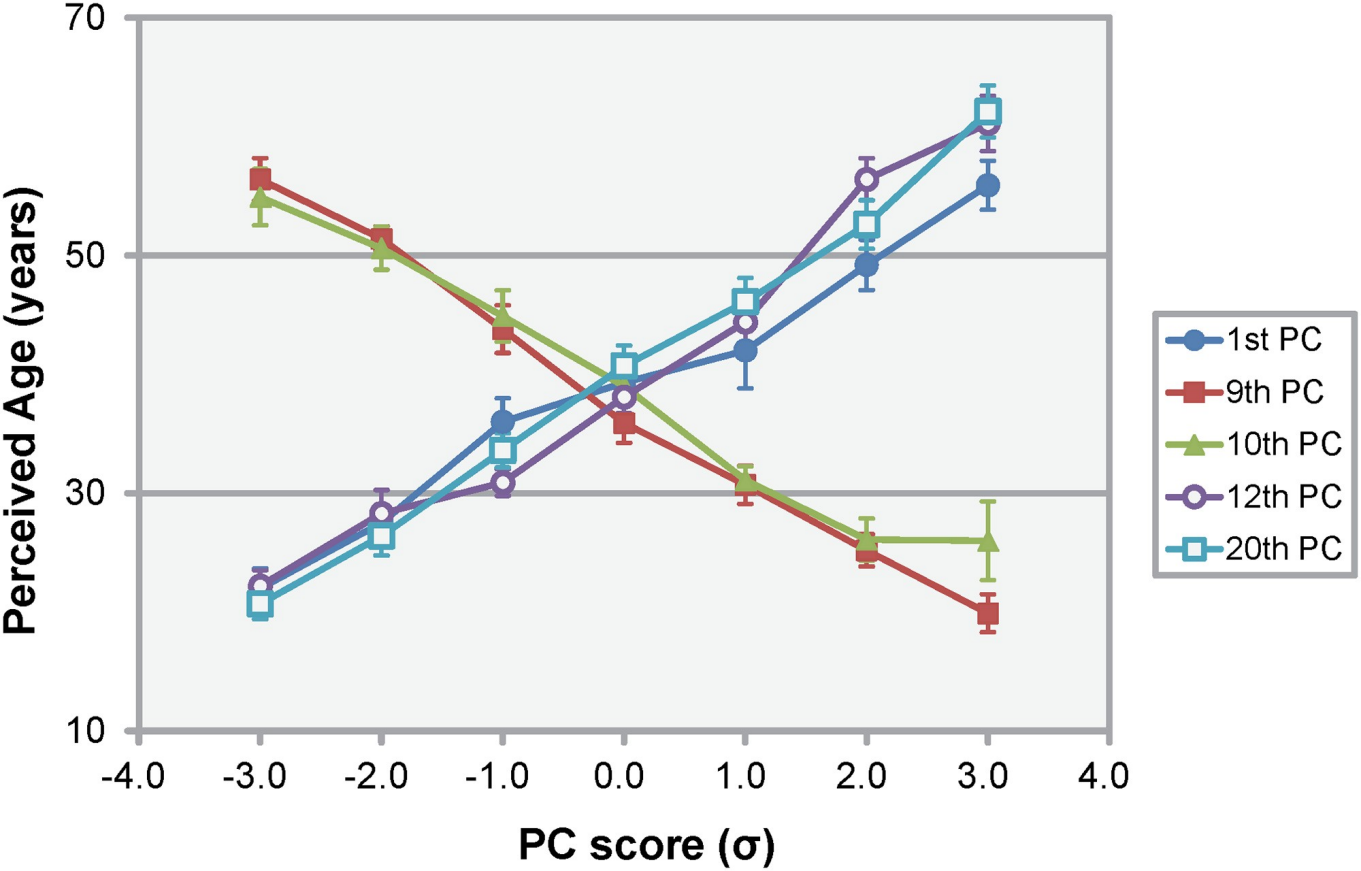
Relationship between the perceived ages of the simulated models^a^ and the age-related principal component (PC) scores^b^. ^a^ Bars represent standard errors. ^b^ Each normalized PC score varied from –3.0 σ to +3.0 σ.

The transformations in these age-related PCs were expected to play a key role in age perception. By contrast, the PCs that did not show a significant correlation with perceived ages may constitute a major cause of individual differences in facial features. To clarify the effects of age-related PCs on age perception, the ages of the simulated polygon models using the five PCs above were estimated. The simulated polygon models were obtained by varying the score of each PC sequentially from –3.0 σ to +3.0 σ (in steps of 1.0 σ). The perceived ages of the simulated polygon models are shown in Fig 4. The internal consistency of the age estimations for the simulated polygon models was calculated using Cronbach’s alpha (α = 0.78). Although only a small number of raters estimated the age of the polygon models, their ratings were consistent. The reliability of the age perception was thus considered sufficient for the purposes of the present study. The results indicate that the perceived ages varied from about 20 to 60 years, depending on the degree of influence of each PC. It is apparent that the perceived ages of the simulated models increased because the expression of age-related PCs in the age-related direction. This variation in perceived age suggests that transformations with these PCs in the age-related direction produced aged faces. When estimating age based on facial appearance, observers have to be sensitive to the whole profile contour (head and face form), as well as facial wrinkling [10]. The perceived age of the polygon models varied, despite having no fine texture on the surface. Therefore, age perception could have been driven by head and face forms. This result supported the findings from previous studies showing that age perception was primarily based on shape cues [16, 17] and revealed that the transformations with five age-related PCs exert a considerable influence on impressions regarding the aging of the face, despite their relatively small contribution ratios. From the transformations with five PCs, the following changes must be related to age perception: the impression of swelling at the bottom of the face (1st PC), slanting eyes (downward, 9th and 12th PCs), the larger aspect ratio of the lips (9th and 12th PCs), smaller eyes (10th and 20th PC), and the greater height between the nose and mouth (20th PC). We regarded these transformations as the key morphologic factors for age perception.

**Fig 5:**
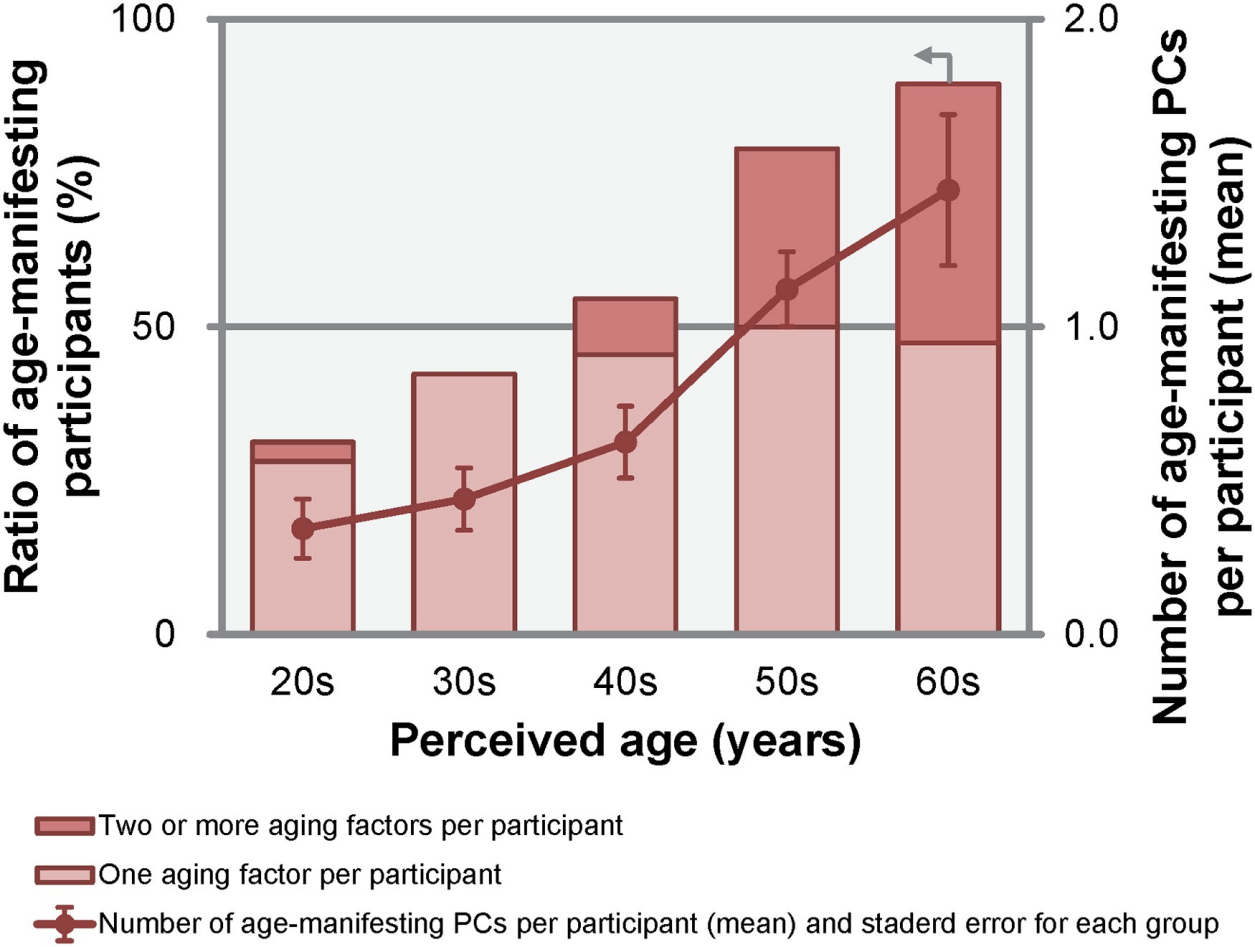
Ratio of age-manifesting participants^a^ in each age cohort and number of age-manifesting principal components (PCs)^b^. ^a^ Participants who had one or more age-related PC score greater than +1.0 σ in the age-related direction. ^b^ Age-related PCs scores greater than +1.0 σ in the age-related direction (bars represent standard errors).

“Age-manifesting PC” refers to age-related PCs with scores greater than +1.0 σ in the age-related direction, and “age-manifesting participants” can be defined as those who have one or more age-manifesting PCs. The relationship between the perceived age and the ratio of age-manifesting participants in each generation, as well as the number of age-manifesting PCs in each participant, is shown in Fig 5. The criterion of +1.0 σ was applied to keep the ratio of age-manifesting participants below 50% in the younger cohorts (20s and 30s), and over 50% in the older cohorts (50s and 60s). As shown in Fig 5, the older the perceived age, the larger the ratio of age-manifesting participants. A one-way ANOVA confirmed that there were statistically significant differences in the number of age-manifesting PCs between the perceived ages (five categories from the 20s through the 60s; *F*(4, 143) = 11.20, *p* < 0.001). Based on the results of the BH procedure for multiple comparisons, the number of age-manifesting PCs for each participant in their 50s and 60s was significantly larger than that for those in their 20s, 30s, and 40s (*p*s < 0.01 and *p*s < 0.001 for the 50s and 60s cohorts, respectively). These findings indicated a tendency for the older participants, especially those in their 50s or above, to have two or more age-manifesting PCs. From these results, we identified these five PCs to be form “aging factors,” the combinations of which may play a decisive role in age perception and aging transformation.

To extract the age-related variations in form, we applied and analyzed relatively late PCs with smaller contribution ratios. Although we examined the correlations between perceived age and all 147 PCs, only the five PCs mentioned above were significant. If only the former PCs with cumulative contribution ratios of 80% or less were adopted, several important morphologic factors with small contribution ratios, such as the 20th PC, could be overlooked.

### 3. Classification of aging-types applying aging factors

We extracted the five aging factors above that were considered key overall for representing the aging transformations of each person. Although several previous studies on age-related changes and features have been based on the analysis of averaged data [19, 50], they failed to shed light on individual differences. Since these were averaged differences, individual differences cannot be described. Therefore, we attempted to classify the participants according to aging factors in order to understand differences in aging transformations between the participants. A cluster analysis (Ward’s method) using these five aging factors revealed that all participants, consisting of all age cohorts (20s to 60s), fell into four groups (the classifications of the participants are listed in the Supporting Information S3 Dataset). The mean perceived ages of groups I, II, III, and IV were 44.6, 43.0, 46.3, and 43.6 years, respectively. A one-way ANOVA was conducted to compare differences in the perceived ages of the four groups. No statistically significant differences were observed between groups in perceived age (*F*(3, 144) = 0.47, *p* = 0.70 [*n*.*s*.]). The component ratios for groups I, II, III, and IV were 26.4, 25.7, 25.7, and 22.3%, respectively (Table 2).

**Table 2:** Results from cluster analysis and characteristic age-related principal components (PCs) for each group.

Group	I	II	III	IV
Number of participants [Component ratio (%)]	39 [26.4]	38 [25.7]	38 [25.7]	33 [22.2]
Mean perceived age (years) [SD]	44.6 [12.9]	43.0 [14.5]	46.3 [11.2]	43.6 [12.6]
Characteristic age-related principal components^a^	9th, 12th	10th, 20th	10th, 9th	9th

^a^ In decreasing order of standardized partial regression coefficient.

The results of the multiple regression analysis conducted to identify the aging factors that play an important role in age perception for each group are shown in S3 Table, and the independent variables that were statistically significant (*p* < 0.05) in each group are listed in Table 2. The results of the above analysis made it clear that there was a specific combination of aging factors in each group: Group I (9th and 12th PCs, in decreasing order of standardized partial regression coefficients); Group II (10th and 20th PCs); Group III (10th and 9th PCs); and Group IV (9th PC). These findings suggest that Japanese women can be classified into four groups according to their manifesting aging factors, which act as cues to age perception. In other words, specific combinations of aging factors in Japanese women can determine the aging path of each individual.

Previous studies have simulated the aging paths specific to each individual using 3D measured forms [24] or 3D forms created from 2D images [23]. The data in those databases were limited to relatively young people, so the studies using these databases were highly suitable for age-related changes involving dynamic and complicated transformations of the cranium during the growth period. However, in middle-aged and elderly generations, aging changes are mainly caused by transformations in soft facial tissue [6–8]. Therefore, it is advantageous to use a database with corresponding generations. As previously mentioned in accordance with Fig 4, the simulations using aging factors in older generations gave impressions of aging without facial textures. It is therefore reasonable to say that the method used in the present study to extract age-related transformations could show aged facial forms across a broad range of generations. On the other hand, several previous studies used not only inter-individual data, but also intra-individual age-progressive data [20, 21]. A database of 3D forms using a homologous polygon model of the same person over several years or longer would be expected to improve the accuracy of these aging methods.

### 4. Age-related transformation in each group

**Fig 6:**
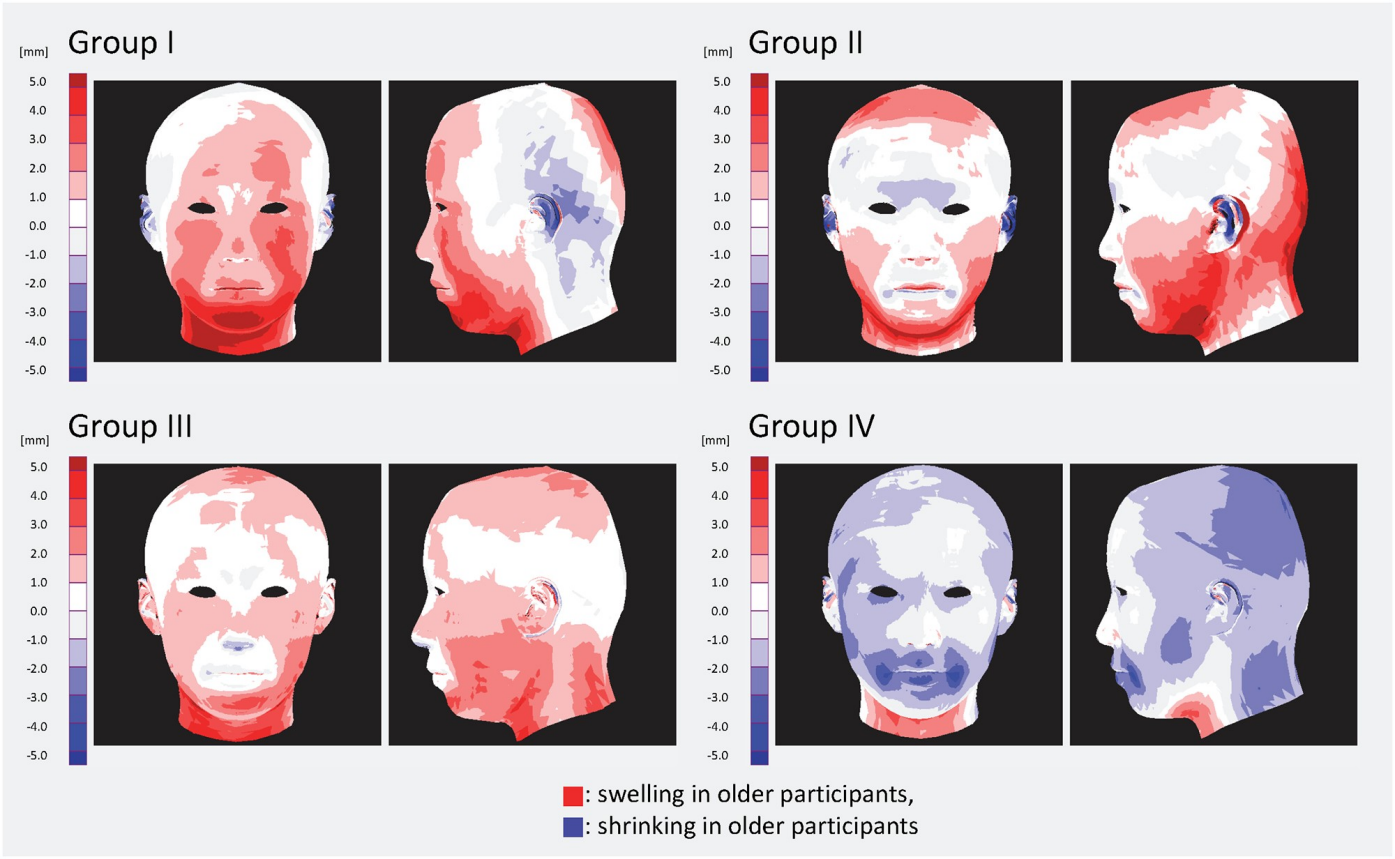
Differences in facial forms between younger and older^a^ participants in each group. ^a^ Participants under and over the mean age of each group.

To clarify age-related transformations in each group, the differences between the average forms of the older and younger participants were measured (Fig 6). The older participants comprised those who were over the mean age of each group, whereas the younger participants comprised those who were under the mean age of each group. The red areas in Fig 6 signify swelling in older participants, while the blue areas signify shrinking. Each group was associated with a specific transformation as follows: faces in group I were larger at the bottom front, faces in group II were larger at the bottom front and side, faces in group III were larger at the bottom front and side, and in contrast to the other three groups, faces in group IV had hollow cheeks. These findings suggest that Japanese women can be classified into four groups according to how they transform with age. These aging transformations can therefore determine the combinations of aging factors that are characteristic of each group. This indicates that aging transformations are not uniform among all people, which suggests the existence of individual aging transformation patterns, and these pattern variations make it difficult to analyze averaged differences between generations [19, 52]. Taken together, these findings strongly suggest that there are specific transformations involving aging factors that could be used as cues for age perception in each group, and that facial features can be classified according to these transformations, namely aging paths.

## Conclusion and future implications

We created a database of actual whole 3D head and face forms of various age groups of Japanese women to clarify facial aging patterns. By statistically analyzing transformations in soft tissue, we could identify cues for age perception as aging factors. From the transformations with five aging factors, the following are proposed as key morphologic factors for aging: the impression of “swelling at the bottom of the face”, “slanting eyes (downward)”, “smaller eyes”, “a larger aspect ratio of the lips”, and “a greater height between the nose and mouth”. We plan to verify these “aging transformations” experimentally in a future study using 3D somatometry. The findings of the present study indicate that there are specific combinations of aging factors in Japanese women, which strongly suggests that facial features can be classified according to their specific age-related transformations. We expect these findings to be helpful in the fields of beauty and aesthetics. For example, by identifying characteristic transformations, priority can be assigned to the parts or areas of the face of each person that will achieve their optimum beauty goals. Individual symptomatic and/or preventive solutions might also be possible in these areas.

Research involving monozygotic twins revealed that the perceived age is a useful biomarker for aging [53]. Although the participants in that study were over 70 years of age, the analysis suggested that the perception of age is affected by not only genetic effects, but also environmental factors and physical status. Therefore, factors other than genetic effects could influence the age-related transformation of soft tissue. In a future study, we plan to analyze age-related changes in the same participants to achieve a more precise aging classification model capable of making assumptions based on environmental and other factors that influence aging transformations.

## Supporting information

S1 Dataset Perceived age rating data for each participant. (XLS)

S2 Dataset Principal component scores for each facial form. (XLS)

S3 Dataset Classification of aging types applying aging factors. (XLS)

S1 Table Landmarks in the head area measured in this study. (DOCX)

S2 Table Landmarks for creating homologous polygon models in the present study. (DOCX)

S3 Table Result of multiple regression analysis for each group. (DOCX)

S1 File Three-dimensional (3D) measurement of the head and face area. (DOCX)
