# Supplementary material for: Facial cues to age perception using three-dimensional analysis
Source: PLoS One. 2019 Feb 13;14(2):e0209639. doi: 10.1371/journal.pone.0209639 (PMC6373935; doi:10.1371/journal.pone.0209639)
Supplement: S1 Table — (DOCX) [file pone.0209639.s004.docx]

S1 Table Landmarks in the head area measured in this study.

| Symbol in Fig. 1 | Anatomical name | Definition | Location^a^ |
| --- | --- | --- | --- |
| v | vertex | the highest point of the head | M |
| - | euryon^b^ | the most lateral point of the temporal region | R/L |
| - | opisthocranion^b^ | the most posterior point in the median plane of the occipital region | M |

^a^ Landmarks on the right and left sides: R/L. Landmark in the median plane: M.

^b^ Landmark did not used for creating homologous polygon models.
