# Supplementary material for: Facial cues to age perception using three-dimensional analysis
Source: PLoS One. 2019 Feb 13;14(2):e0209639. doi: 10.1371/journal.pone.0209639 (PMC6373935; doi:10.1371/journal.pone.0209639)
Supplement: S2 Table — (DOCX) [file pone.0209639.s005.docx]

S2 Table Landmarks for creating homologous polygon models in the present study.

| Abbreviation in Fig. 1 | Anatomical name | Definition | Location^a^ |
| --- | --- | --- | --- |
| en | entocanthion | the medial corner of the eye | R/L |
| ex | ectocanthion | the lateral corner of the eye | R/L |
| ps | palpebrale superius | the center of the upper eyelid margin | R/L |
| ps’^b^ | - | the center of the upper eyelid crease | R/L |
| pi | palpebrale inferius | the center of the lower eyelid margin | R/L |
| al | alare | the most lateral points of the wings of the nose | R/L |
| sn | subnasale | the most inferior point of nose in the median plane | M |
| ls | labrale superius | the top of upper lip in the median plane | M |
| sto | stomion | the median point of oral slit with lips closed | M |
| li | labrale inferius | the bottom of lower lip in the median plane | M |
| ch | cheilion | the corner of the mouth. | R/L |
| tr | trichion | the midpoint of the hairline at the top of the forehead | M |
| pa | postaurale | the most posterior and lateral point of the external ear | R/L |
| prn | pronasale | the tip of the nose | M |
| cph | crista philtri | the top end of the upper lip | R/L |
| ft^c, d^ | frontotemporale | the point in the upper area of the peak of the arch of each eyebrow | R/L |
| zy^c, d^ | zygion | the most lateral point on the zygomatic arch (cheek bone) | R/L |
| v^e^ | vertex | the highest point of the head | M |
| t | tragion | the notch just above the tragus | R/L |
| a1 | additional landmark-1 | the most prominent point of the forehead area (around frontal tuber) | R/L |
| a2 | additional landmark-2 | the most prominent point in the upper area of the inner end of the eyebrow | R/L |
| a3 | additional landmark-3 | the saddle point of the lateral part of ectocanthion | R/L |
| a4 | additional landmark-4 | the point of the most lateral point in the cheek part of the contour line in z-axis which passes along both ectocanthions | R/L |
| a5*^f^ | additional landmark-5 | the most prominent point near gonion (the most inferior, posterior, and lateral point on the angle of the mandible [lower jawbone]) | R/L |
| a6 | additional landmark-6 | the most prominent (anterior) points in the front chin area*^3^ | R/L |
| or*^3, c, d, g^ | orbitale | the lowest point on the lower edge of the orbit (eye socket) | R/L |

^a^ Landmarks in the right and left sides: R/L. Landmark in the median plane: M.

^b^ If the upper eyelid was single-edged (without visible fold), we marked the same point as palpebrale superius.

^c^ Landmark located by palpation before 3D measurement.

^d^ Landmarks those are only locatable by palpation. These points were marked before 3D measurement using stickers.

^e^ Landmark is also listed in S1 Table..

^f^ If an apex was in the median plane, we marked two points horizontally beside the apex.

^g^ Landmark did not used for creating homologous polygon models.
