# Supplementary material for: Facial cues to age perception using three-dimensional analysis
Source: PLoS One. 2019 Feb 13;14(2):e0209639. doi: 10.1371/journal.pone.0209639 (PMC6373935; doi:10.1371/journal.pone.0209639)
Supplement: S3 Table — (DOCX) [file pone.0209639.s006.docx]

S3 Table Result of multiple regression analysis^a^ for each group.

| Group | PCs | Partial regression coefficient | Standard partial regression coefficient | F-value | p-value | Standard error | Variance inflation factor |
| --- | --- | --- | --- | --- | --- | --- | --- |
| I | 1st PC | 0.054 | 0.226 | 1.833 | 0.185 | 0.040 | 1.90 |
|  | 9th PC | -0.352 | -0.469 | 10.157 | 0.003 | 0.111 | 1.47 |
|  | 10th PC | -0.047 | -0.065 | 0.173 | 0.680 | 0.112 | 1.67 |
|  | 12th PC | 0.359 | 0.300 | 5.464 | 0.026 | 0.154 | 1.12 |
|  | 20th PC | 0.449 | 0.269 | 3.912 | 0.056 | 0.227 | 1.26 |
|  | constant term | 42.692 |  | 161.684 | 0.000 | 3.357 |  |
| II | 1st PC | 0.051 | 0.210 | 3.088 | 0.088 | 0.029 | 1.16 |
|  | 9th PC | -0.156 | -0.185 | 2.702 | 0.110 | 0.095 | 1.02 |
|  | 10th PC | -0.228 | -0.298 | 5.256 | 0.029 | 0.099 | 1.37 |
|  | 12th PC | 0.224 | 0.212 | 2.557 | 0.120 | 0.140 | 1.41 |
|  | 20th PC | 0.420 | 0.291 | 4.430 | 0.043 | 0.199 | 1.54 |
|  | constant term | 41.457 |  | 170.438 | 0.000 | 3.176 |  |
| III | 1st PC | 0.035 | 0.150 | 1.263 | 0.269 | 0.031 | 1.13 |
|  | 9th PC | -0.191 | -0.311 | 5.372 | 0.027 | 0.082 | 1.15 |
|  | 10th PC | -0.280 | -0.370 | 7.024 | 0.012 | 0.106 | 1.24 |
|  | 12th PC | 0.213 | 0.292 | 3.433 | 0.073 | 0.115 | 1.58 |
|  | 20th PC | 0.321 | 0.253 | 2.825 | 0.103 | 0.191 | 1.45 |
|  | constant term | 45.716 |  | 196.247 | 0.000 | 3.263 |  |
| IV | 1st PC | 0.069 | 0.206 | 1.722 | 0.201 | 0.053 | 1.57 |
|  | 9th PC | -0.416 | -0.529 | 9.065 | 0.006 | 0.138 | 1.96 |
|  | 10th PC | -0.287 | -0.259 | 3.604 | 0.068 | 0.151 | 1.18 |
|  | 12th PC | 0.363 | 0.305 | 3.483 | 0.073 | 0.195 | 1.69 |
|  | 20th PC | 0.324 | 0.210 | 1.709 | 0.202 | 0.248 | 1.63 |
|  | constant term | 41.741 |  | 68.495 | 0.000 | 5.044 |  |

^a^ Dependent variable: perceived age, independent variable: the PC scores of the five aging factors
