# Supplementary material for: Facial cues to age perception using three-dimensional analysis
Source: PLoS One. 2019 Feb 13;14(2):e0209639. doi: 10.1371/journal.pone.0209639 (PMC6373935; doi:10.1371/journal.pone.0209639)
Supplement: S1 File — (DOCX) [file pone.0209639.s007.docx]

# Supporting information

## 1. Three-dimensional (3D) measurement of the head and face area

### 1-1 Landmarks in the head area

We measured 16 to 24 coordinates, including the following four landmarks: vertex, euryons (right and left sides [R/L]), and the opisthocranion (S1 Table).

S1 Table Landmarks in the head area measured in this study.

| Symbol in Fig. 1 | Anatomical name | Definition | Location*^1^ |
| --- | --- | --- | --- |
| v | vertex | the highest point of the head | M |
| - | euryon*^2^ | the most lateral point of the temporal region | R/L |
| - | opisthocranion*^2^ | the most posterior point in the median plane of the occipital region | M |

*^1^ Landmarks on the right and left sides: R/L. Landmark in the median plane: M.

*^2^ Landmark did not used for creating homologous polygon models.

### 1-2 Landmarks for identifying the Frankfurt plane and creating homologous polygon models

The Frankfurt plane, which is defined as the plane that passes through two tragia and one orbitale (S2 Table), served as the anatomical reference position of the head and face area. Both orbitales (or), frontotemporales (fr), and zygions (zy) are only locatable by palpation, so these points were marked before 3D measurement using stickers.

We described the landmarks for creating homologous polygon models in Fig. 1 and S2 Table.

S2 Table Landmarks for creating homologous polygon models in the present study.

| Abbreviation in Fig. 1 | Anatomical name | Definition | Location*^1^ |
| --- | --- | --- | --- |
| en | entocanthion | the medial corner of the eye | R/L |
| ex | ectocanthion | the lateral corner of the eye | R/L |
| ps | palpebrale superius | the center of the upper eyelid margin | R/L |
| ps’*^2^ | - | the center of the upper eyelid crease | R/L |
| pi | palpebrale inferius | the center of the lower eyelid margin | R/L |
| al | alare | the most lateral points of the wings of the nose | R/L |
| sn | subnasale | the most inferior point of nose in the median plane | M |
| ls | labrale superius | the top of upper lip in the median plane | M |
| sto | stomion | the median point of oral slit with lips closed | M |
| li | labrale inferius | the bottom of lower lip in the median plane | M |
| ch | cheilion | the corner of the mouth. | R/L |
| tr | trichion | the midpoint of the hairline at the top of the forehead | M |
| pa | postaurale | the most posterior and lateral point of the external ear | R/L |
| prn | pronasale | the tip of the nose | M |
| cph | crista philtri | the top end of the upper lip | R/L |
| ft*^3^ | frontotemporale | the point in the upper area of the peak of the arch of each eyebrow | R/L |
| zy*^3^ | zygion | the most lateral point on the zygomatic arch (cheek bone) | R/L |
| v*^4^ | vertex | the highest point of the head | M |
| t | tragion | the notch just above the tragus | R/L |
| a1 | additional landmark-1 | the most prominent point of the forehead area (around frontal tuber) | R/L |
| a2 | additional landmark-2 | the most prominent point in the upper area of the inner end of the eyebrow | R/L |
| a3 | additional landmark-3 | the saddle point of the lateral part of ectocanthion | R/L |
| a4 | additional landmark-4 | the point of the most lateral point in the cheek part of the contour line in z-axis which passes along both ectocanthions | R/L |
| a5*^5^ | additional landmark-5 | the most prominent point near gonion (the most inferior, posterior, and lateral point on the angle of the mandible [lower jawbone]) | R/L |
| a6 | additional landmark-6 | the most prominent (anterior) points in the front chin area*^3^ | R/L |
| or*^3, 6^ | orbitale | the lowest point on the lower edge of the orbit (eye socket) | R/L |

*^1^ Landmarks in the right and left sides: R/L. Landmark in the median plane: M.

*^2^ If the upper eyelid was single-edged (without visible fold), we marked the same point as palpebrale superius.

*^3^ Landmark located by palpation before 3D measurement.

*^4^ Landmark is also listed in S1 Table.

*^5^ If an apex was in the median plane, we marked two points horizontally beside the apex.

*^6^ Landmark did not used for creating homologous polygon models.
